# Eyes on the past: Gaze stability differs between temporal expectation and temporal attention

**DOI:** 10.1167/jov.25.4.11

**Published:** 2025-04-16

**Authors:** Aysun Duyar, Marisa Carrasco

**Affiliations:** 1 Department of Psychology, New York University, New York, NY, USA; 2 Center for Neural Science, New York University, New York, NY, USA

**Keywords:** temporal expectation, temporal attention, sequential effects, microsaccades

## Abstract

Does the timing of a preceding visual event affect when people deploy attention in the future? Temporal expectation and temporal attention are two distinct processes that interact at the behavioral and neural levels, improving performance and gaze stability. The preceding foreperiod—the interval between the preparatory signal and stimulus onset in the previous trial—modulates expectation at the behavioral and oculomotor levels. Here, we investigated whether the preceding foreperiod also modulates the effects of temporal attention and whether such effects interact with expectation. We found that, regardless of whether the stimulus occurred earlier than, later than, or at the expected moment in the preceding foreperiod, temporal attention improved performance and accelerated gaze stability onset and offset consistently by shifting microsaccade timing. However, overall, only with expected preceding foreperiods, attention inhibited microsaccade rates. Moreover, late preceding foreperiods weakened the expectation effects on microsaccade rates, but this weakening was overridden by attention. Altogether, these findings reveal that the oculomotor system's flexibility does not translate to performance, and suggest that, although selection history can be used as one of the sources of expectation in subsequent trials, it does not necessarily determine, strengthen, or guide attentional deployment.

## Introduction

The visual environment is in a continuous state of flux, surpassing the brain's processing capabilities (Shapiro, 2001). Sequential effects emerge as a consequence of this capacity limitation; many perceptual judgements are biased toward previous stimuli in vision (Fischer & Whitney, 2014; Pascucci et al., 2023), audition (Motala, Zhang, & Alais, 2020), and olfaction (van der Burg, Toet, Brouwer, & van Erp, 2022). The brain's limited temporal processing capacity emphasizes the necessity for optimizing its resources to navigate the temporal constraints imposed by its own biological architecture (Nobre & van Ede, 2023). Temporal expectation (from now on, expectation)—the ability to formulate predictions about when a visual event is likely to occur—and temporal attention (from now on, attention)—the ability to prioritize and selectively process the specific time points based on behavioral relevance regardless of its predictability—are fundamental cognitive processes that act together to distribute these limited resources within a time window (Denison, 2024; Denison, Heeger, & Carrasco, 2017; Denison, Carrasco, & Heeger, 2021; Denison, Tian, Heeger, & Carrasco, 2024; Duyar, Denison & Carrasco, 2023; Nobre & van Ede, 2023; Todorovic, Schoffelen, van Ede, Maris, & de Lange, 2015; Zhu, Tian, Carrasco, & Denison, 2024). Moreover, these two processes interact when modulating visual performance (Duyar, Ren, & Carrasco, 2024) and neural responses (Todorovic et al., 2015). Together, expectation and attention improve visual processing based on the probabilities and behavioral goals associated with the dynamic visual environment. However, it is unknown whether there are sequential effects of event timing on the interplay between temporal attention and expectation. Here we investigate potential sequential effects in behavior and oculomotor responses.

Temporal uncertainty—the unpredictability regarding when events may occur—impairs visual processing (Rolke & Hoffman, 2007). Temporal expectation, indexed by modulations in accuracy, discriminability, response time, and visual representations (Coull & Nobre, 2008; Cravo, Rohenkohl, Wyart, & Nobre, 2013; Nobre, Correa, & Coull, 2007; Nobre & van Ede, 2023; Rohenkohl, Cravo, Wyart, & Nobre, 2012; Rohenkohl, Gould, Pessoa, & Nobre, 2014; Shalev & Nobre, 2022; van den Brink, Murphy, Desender, de Ru, & Nieuwenhuis, 2021; Vangkilde, Coull, & Bundesen, 2012), improves visual performance. Directing attention to a time point in which the behaviorally relevant stimulus is expected to appear further improves performance (Correa, Lupianez, & Tudela, 2005; Denison et al., 2021; Denison et al., 2017; Duyar et al., 2024; Fernández, Denison, & Carrasco, 2019; Griffin, Miniussi, & Nobre, 2001; Jing et al., 2023). These benefits at the attended moments emerge at the expense of impairments at unattended but expected moments, resulting in attentional tradeoffs (Denison et al., 2017; Denison et al., 2021; Palmieri & Carrasco, 2024).

Research on temporal orienting, which investigates how cognitive resources are distributed across time, rarely differentiates between expectation and attention in their definitions and manipulations. Some studies have shown that attention modulates performance beyond expectation (Denison et al., 2017; Denison et al., 2021; Denison, Yuval-Greenberg, & Carrasco, 2019; Fernández et al., 2019; Jing et al., 2023; Palmieri & Carrasco, 2024). By keeping expectations constant, these studies have isolated the role of attention.

Both expectation and attention also modulate oculomotor behavior. Although visual perception and eye movements are considered to be tightly linked, perceptual reports and different types of eye movements have been dissociated (e.g., Glasser & Tadin, 2014; Spering & Carrasco, 2012; Spering, Pomplun, & Carrasco, 2010; Vetter, Badde, Phelps, & Carrasco, 2019). Our eyes move even during fixation. Microsaccades, the fastest and largest of these fixational eye movements, with 1 degree or less of visual angle gaze displacements at a frequency of approximately 1 to 2 Hz, provide a continuous measure of perceptual and cognitive processing throughout experimental trials (Martinez-Conde, Macknik, Troncoso, & Hubel, 2009; Martinez-Conde, Otero-Millan, & Macknik, 2013; Rolfs, 2009; Rucci & Poletti, 2015). Perceptual performance drops if a microsaccade is executed around the presentation of a brief stimulus (Beeler, 1967; Hafed & Krauzlis, 2010; Zuber & Stark, 1966). Accordingly, the microsaccade rate decreases in anticipation of a brief stimulus in visual (e.g., Betta & Turatto, 2006; Dankner, Shalev, Carrasco, & Yuval-Greenberg, 2017; Fried et al., 2014; Hung & Carrasco, 2022; Tal-Perry & Yuval-Greenberg, 2020), auditory (Abeles, Amit, Tal-Perry, Carrasco, & Yuval-Greenberg, 2020) and tactile (Badde, Myers, Yuval-Greenberg, & Carrasco, 2020) perception, often leading to improvements in perceptual performance.

When attention is deployed to an expected stimulus, it further suppresses microsaccade rates in the pre-stimulus window (Denison et al., 2019; Palmieri, Fernández & Carrasco, 2023). This suppression, however, is not necessarily linked to attentional benefits on visual performance (Palmieri et al., 2023), indicating that attention can distinctly modulate oculomotor dynamics and visual performance. In addition, the last microsaccade before and the first microsaccade after the stimulus are executed at an earlier time point with attention than mere expectation, helping to stabilize fixation at the most relevant moments (Denison et al., 2019).

Different temporal structures enable distinct types of expectation (Nobre & van Ede, 2018). The foreperiod—the interval between the preparatory signal and the stimulus onset—and preceding foreperiod—the corresponding interval in the previous trial—are two types of temporal structures (Capizzi, Correa, Wojtowicz, & Rafal, 2015; Tal-Perry & Yuval-Greenberg, 2023). The foreperiod affects expectations by improving performance and microsaccades: As the foreperiod lengthens and the stimulus is delayed (hazard rate), reaction times (RTs) decrease, discriminability improves (Duyar et al., 2024; Janssen & Shadlen, 2005; Los, 2010; Luce, 1991; Schoffelen, Oostenveld, & Fries, 2005), and microsaccade rates diminish (Abeles et al., 2020; Amit, Abeles, Carrasco, & Yuval-Greenberg, 2019; Badde et al., 2020; Dankner et al., 2017). Furthermore, the preceding foreperiod modulates these effects (e.g., Vallesi & Shallice, 2007): As the preceding foreperiod lengthens, the RT advantage disappears (Steinborn, Rolke, Bratzke, & Ulrich, 2008) and microsaccade inhibition becomes weaker (Tal-Perry & Yuval-Greenberg, 2023).

In the real world, external noise leads to stochasticity in the timing of events, and strategic allocation of attention relies on the ability to anticipate when behaviorally relevant events are likely to occur (Capizzi, Martín-Signes, Coull, Chica, & Charras, 2023; Nobre & van Ede, 2018; Nobre & van Ede, 2023). However, the interplay between attention and expectation under temporal uncertainty has rarely been investigated (Duyar et al., 2023; Duyar et al., 2024; Todorovic et al., 2015). Temporal uncertainty can be quantified and manipulated by systematically increasing the variability of event onsets within a given window (Rolke & Hofmann, 2007). This variability influences the allocation of attention. As it increases, attentional benefits on performance decrease at the expected moment—the time point with the greatest probability of target occurrence. Furthermore, when variability is high, attention tends to be allocated to earlier than the expected moment (Duyar et al., 2024).

The extent of flexibility in attentional deployment under uncertainty–when the target is expected to occur in a temporal window rather than at a specific moment–remains unexplored. Here, we investigated whether attention is automatically allocated to earlier moments of the temporal window or if recently experienced stimulus onsets facilitate trial-to-trial allocation adjustments of attention. Considering the impact of expectations on attention (Duyar et al., 2023; Duyar et al., 2024), the effects of preceding foreperiods on expectations (Capizzi et al., 2015; Los, 2010; Tal-Perry & Yuval-Greenberg, 2022; Tal-Perry & Yuval-Greenberg, 2023), and perception and eye movements dissociations (for reviews, see Carrasco & Spering, 2024; Spering & Carrasco, 2015), we asked whether preceding foreperiods modulate the allocation of attention under uncertainty and whether such effects would be manifested similarly in performance and microsaccades. The findings provide insight into how expectations about stimulus timing are incorporated into the cognitive control of attentional orienting and temporal selection.

We hypothesized that the preceding foreperiod would affect the allocation of attention under temporal uncertainty. If the stimuli appeared at a late moment in the preceding trial, observers might not be able to attend to an early moment in the current trial, resulting in greater attentional benefits on visual performance at late moments and a delayed onset of the inhibition and rebound of microsaccades around the stimulus onset. Accordingly, microsaccade rates could also decrease more slowly. Moreover, preceding foreperiod could either jointly modulate performance and microsaccade dynamics, consistent with similar effects in perception and eye movements (for reviews, see Kowler, 2011; Schütz, Braun, & Gegenfurtner, 2011; Spering & Montagnini, 2011) or exert independent effects, consistent with dissociations between perception and the oculomotor system (for reviews, see Carrasco & Spering, 2024; Spering & Carrasco, 2015).

## Methods

### Dataset

We reanalyzed behavioral and eye-tracking data collected in a recent study investigating temporal attention modulations on visual performance under temporal uncertainty by Duyar et al. (2024). All observers, apparatus, stimuli, as well as the experimental procedure were identical to those previously reported. In that study, there were four levels of temporal precision, manipulated by stimulus timing variability within a session. For this study, we only included the two lowest precision conditions (42% wide and 33% uniform) in the analysis, as in the “hazard rate” section of the previous study (Duyar et al., 2024). To analyze whether temporal attention effects on visual performance and oculomotor dynamics are modulated by preceding foreperiods, we focused on these two temporal precision conditions in which there was high temporal variability and enough number of trials for each type of preceding foreperiod (Early_N-1_, Expected_N-1_, and Late_N-1_).

### Observers

The observers were the same 16 observers (10 females, six males, aged between 22 and 34 years). We determined the number of observers by conducting a power analysis using G*Power software (Faul, Erdfelder, Lang, & Buchner, 2007). We computed the necessary number of observers to achieve 80% power for a potential interaction between temporal attention and expectation, using the effect size of *η_G_*^2^ = 0.14, reported in a previous temporal attention study (Fernández et al., 2019). All participants provided their written consent and had normal or corrected-to-normal vision. The experimental methods complied with the Helsinki Declaration and received approval from the New York University Institutional Review Board.

### Apparatus

Stimuli were generated using an Apple iMac (3.06 GHz, Intel Core 2 Duo) and MATLAB 2012b (Mathworks, Natick, MA), along with the Psychophysics Toolbox (Brainard, 1997; Kleiner et al., 2007), displayed on a color-calibrated CRT monitor (1,280 × 960 resolution, 100 Hz refresh rate). Observers were seated 57 cm away from the screen; their heads were stabilized with a chinrest. Eye position was monitored and recorded using the Eyelink 1000 eye tracker (SR Research, Ottawa, Ontario, Canada) monocularly using a 1,000-Hz sampling rate. If a participant blinked or moved their eye more than 1° from the screen's center during the critical time window (between the pre-cue and the response cue in each trial), the trial was automatically aborted and repeated at the end of the block.

### Experimental protocol

**Figure 1.**
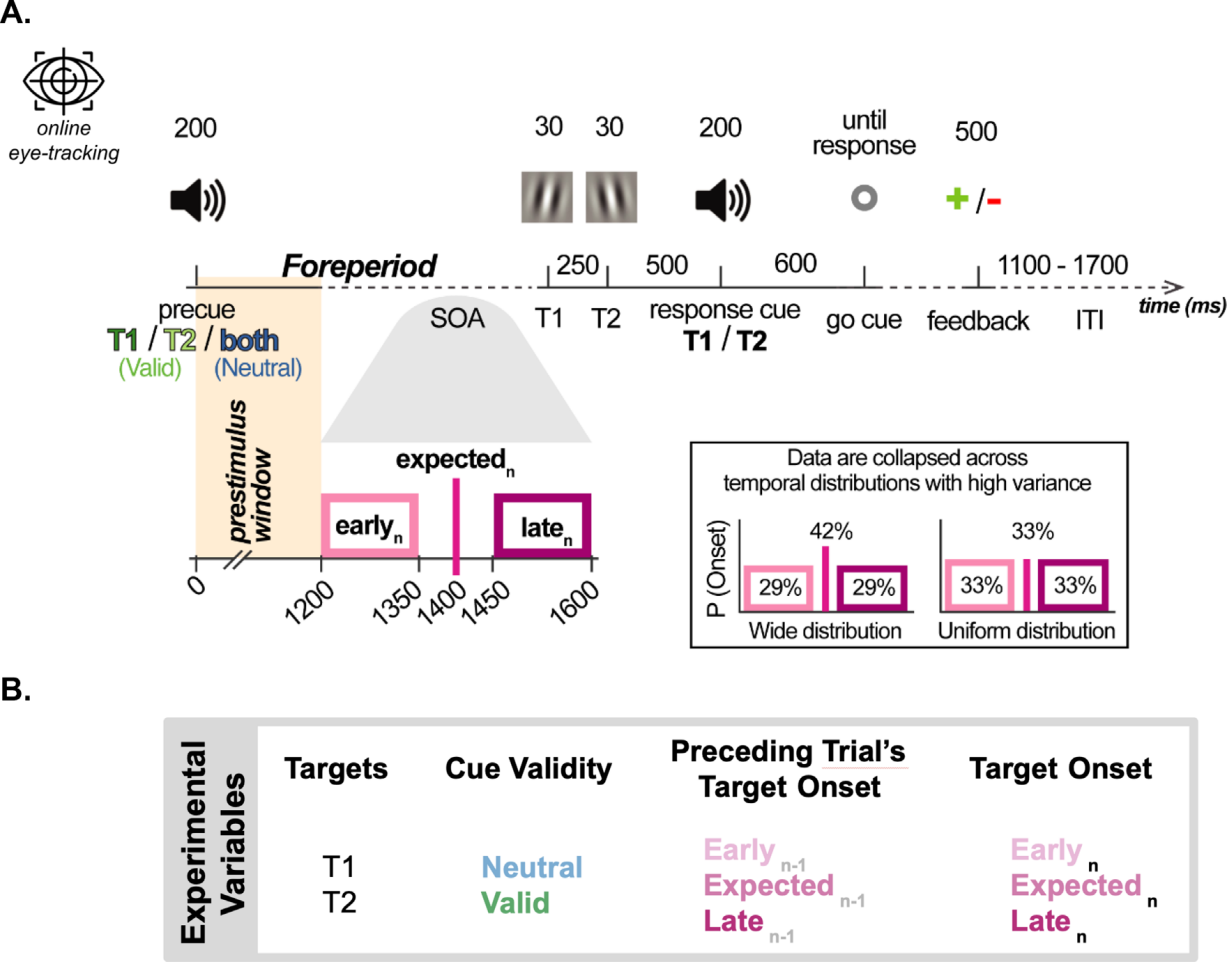
(**A**) Psychophysical procedure assessed visual performance at attended and unattended moments, with a variable stimulus timing. The exact timing of the stimulus onset varied across sessions, and we analyzed the data from the sessions with highest variance, “Uniform” and “Wide” temporal distributions. Expected moment is the time point when the stimuli could appear with highest probability (mean point of the temporal distributions). Stimuli could appear earlier or later than the expected time. For microsaccades, we analyzed the “pre-stimulus window”: the interval between the pre-cue and the earliest possible stimulus onset. Foreperiod is defined as the interval between the pre-cue and T1, and the preceding foreperiod is this interval in the most recent trial. (**B**) Summary of experimental variables. There were two targets in each trial, and performance was tested separately for T1 and T2 indicated by the response cue. The pre-cue was either valid or neutral to probe temporal attention. Temporal expectation was manipulated via target timing. Preceding trial's target onset corresponds with preceding foreperiod, and the target onset corresponds to the current foreperiod.

Two oriented Gabors were presented at the fovea sequentially in each trial, and observers performed a two-alternative forced choice orientation discrimination task (Figure 1A).

In the beginning of each trial, an auditory pre-cue instructed participants to attend to either the first target (T1), the second target (T2), or both (neutral). Each target was shown for 30 ms with a stimulus onset asynchrony of 250 ms. The response cue was presented 500 ms after the T2 onset, instructing observers to report the orientation of the first (T1), or the second target (T2), choosing either clockwise or counterclockwise with respect to the horizontal or vertical reference using the keyboard. Participants responded after the go-cue (800 ms after the response cue onset), signaled by a brightness change of the fixation point.

In neutral trials, the response cue indicated the first or second Gabor patch with equal probability. In valid trials, the pre-cue was 100% accurate, and the response cue consistently matched the pre-cue. Feedback was given after the observer's response for 500 ms, a red minus for incorrect responses and a green plus for correct responses. Participants were advised to prioritize accuracy rather than speed, and there was no time constraint on their response. The intertrial interval varied from 1,100 to 1,700 ms.

The onset of the targets with respect to the pre-cue (pre-cue-T1 stimulus onset asynchrony) was randomized throughout the experiment. T1 onset varied between 1,200 and 1,600 ms and T2 between 1,450 and 1,850 ms after the pre-cue, with the most possible onsets occurring at 1,400 ms for T1 and 1,650 ms for T2 after the pre-cue onset. As a result, the targets could appear either earlier, at, or later than the expected time point. The Early and Late windows for both targets spanned 150 ms, and the timings of Early and Late targets were randomly selected from this uniform distribution with 1-ms steps. Within each block, we ensured that there were an equal number of trials in both halves of these windows (i.e., an equal number of onsets for T1_Early_ ∈ [1,200, 1,275] and T1_Early_ ∈ [1,275, 1,350], and for T1_Late_ ∈ [1,500, 1,575] and T1_Late_ ∈ [1,575, 1,650]) (Figure 1A). This design enabled us to assess performance at the moments both earlier and later than the expected moment.

There were four experimental factors in this study: target, temporal attention, temporal expectation, and preceding foreperiod (Figure 1B).

We titrated the neutral performance level at the expected moments (1,400 ms for T1 and 1,650 ms for T2 following the pre-cue) to a 75% accuracy rate for each target independently, across horizontal and vertical orientations, before starting each session. The initial tilt threshold for each session was established using the Best parameter estimation by sequential testing technique (Lieberman & Pentland, 1982), and this threshold was adjusted after each block as needed to maintain the target neutral accuracy close to 75%.

The trial sequence was randomized for each session, and the sequence of sessions for different temporal precision levels varied across participants. Each participant completed between five or six sessions, amounting to a total of 5.3 on average per participant (ranging between 2,551 and 3,120 trials per participant). Eye tracking data from nine sessions (of 85) were corrupted owing to unforeseen technical issues with the equipment; thus, we analyzed the eye tracking data from 2,252 trials on average per participant (ranging between 1,527 and 3,081 trials).

### Stimuli

Stimuli were displayed on a uniform medium gray background. A fixation circle with a diameter of 0.15° of visual angle was presented at the center of the screen. The placeholders were four small black circles, each 0.2° in diameter, positioned at the corners of an imaginary square with a side length of 2.2°, and remained on the screen throughout the trials to minimize spatial uncertainty.

The target stimuli were 100% contrast, Gaussian-windowed sinusoidal gratings (standard deviation = 0.3°, 4 cycles per degree, and random phase). The gratings were oriented either clockwise or counterclockwise from the vertical or horizontal axis. The degree of tilt was titrated independently for each observer and each target interval to attain 75% accuracy at the expected timing in the neutral trials.

The pre-cue and the response cue were auditory stimuli, presented through speakers. The attentional pre-cue was a 200-ms auditory tone, either a sinusoidal wave or a complex waveform composed of sinusoidal waves ranging from 50 to 400 Hz in frequency. A 800 Hz high-frequency sinusoidal tone indicated the first target (T1), a 440 Hz low-frequency tone indicated the second target (T2), and a complex tone was uninformative regarding the target (neutral pre-cue). The same tones, excluding the neutral tone, were also used at the end of the trial as a response cue to indicate that observers should respond with regard to the stimulus presented in either T1 or T2.

### Behavioral data analysis

The discriminability index, d′, was calculated as *z* (hit rate) − *z* (false alarm rate) (e.g., Fernández & Carrasco, 2020; Zhang, Morrone, & Alais, 2019). To prevent infinite values in the calculation of d′, a correction was applied, adding 0.5 to the count of hits, misses, correct rejections, and false alarms (Brown & White, 2005; Hautus, 1995).

Median RTs from the go-cue for correct responses were used in the analysis.

We evaluated behavioral performance for five experimental factors: cue validity (Neutral and Valid), target (T1 and T2), current foreperiod timing (Early_N_, Expected_N_, and Late_N_), preceding foreperiod (Early_N-1_, Expected_N-1_, and Late_N-1_) and preceding performance (Correct_N-1_ and Incorrect_N-1_); the last two factors were not analyzed previously (Duyar et al., 2024).

Data analysis was performed using R software (version 4.2.3; R Core Team, 2020), and repeated measures analysis of variance (ANOVA) was conducted using the ezANOVA package (version 4.4–0; Lawrence, 2016) and *η_G_*^2^ was provided for all F tests. As a rule of thumb, *η_G_*^2^ = 0.01 is interpreted as a small effect, *η_G_*^2^ = 0.06 as a medium effect, and *η_G_*^2^ = 0.14 as a large effect (Cohen, 1988), although this metric is more appropriate to compare across effect sizes in different studies (Lakens, 2013; Thompson, 2007). Greenhouse–Geisser corrections were applied when Mauchly's test indicated sphericity was violated. To report effect sizes, Cohen's d was computed for significant differences revealed by post hoc two-tailed *t* tests, interpreted as *d* = 0.2 as a small effect, *d* = 0.5 as a medium effect, and *d* = 0.8 as a large effect (Cohen, 1988; Fritz, Morris, & Richler, 2012).

As a post hoc analysis, we also performed Bayesian statistics using the “BayesFactor” package in R. The Bayes factor (BF_10_) for main effects and interaction effects in the repeated measures ANOVA design were computed by comparing the full model (H_1_) against the restricted model (H_0_) in which the effect of interest was excluded from the full model (Rouder, Morey, Verhagen, Swagman, & Wagenmakers, 2017). BF_10_ smaller than 1 supports the absence of an effect; values of 1 to 3, 3 to 10, 10 to 30, 30 to 100, and greater than 100 indicate anecdotal, moderate, strong, very strong, and extreme evidence for the presence of an effect (Kass & Raftery, 1995; Lee & Wagenmakers, 2014).

### Microsaccade preprocessing and analysis

#### Preprocessing

We performed the preprocessing of eye tracking data and microsaccade analyses using MATLAB. We used a standard velocity-based microsaccade detection algorithm to extract microsaccade (Engbert & Kliegl, 2003). For each eye-tracking sample, eye position displacement was mapped to a two-dimensional velocity space. Saccades were defined as at least 6 ms duration of consecutive velocities greater than 6 standard deviations from the average velocity within each trial, and microsaccade were defined as saccades smaller than 1 degree of visual angle.

#### Online monitoring and experimental conditions throughout the trial

Microsaccades provide an online measure across the trial. Owing to the fact that some of the experimental conditions unfold later in the trial, we used different factors in microsaccade analysis than those we used to analyze behavioral performance. Instead of the cue validity and target (Neutral vs Valid for T1 and T2), which are determined by the response cue at the end of the trial, we analyze online attentional modulations based on the pre-cue (T1, T2, and Neutral). Similarly, instead of the current foreperiod (Early_N_, Expected_N_, and Late_N_), which is unknown to the observer before the stimulus occurs, we collapse across trials with different timings in the pre-stimulus window. Consequently, we have two factors: pre-cue (T1, T2, and Neutral) and preceding foreperiod (Early_N-1_, Expected_N-1_, and Late_N-1_) in the pre-stimulus window. We defined the pre-stimulus window as the interval from the pre-cue onset to 1,200 ms later, which marks the earliest possible stimulus onset (see Figure 1A).

#### Microsaccade rate analysis

Microsaccade rates were calculated separately for each experimental condition for each observer. We calculated the rate time-course with respect to the pre-cue, and then implemented temporal averaging by using an exponential window causal filter, and lastly smoothing using a moving Gaussian window (σ = 100 ms). This window size is common, within the 50 to 200 ms range typically implemented in microsaccade rate analysis with cluster-based permutation tests (e.g., Laubrock, Engbert, & Kliegl, 2005; Denison et al., 2019; Badde et al., 2020; Otero-Millan, Macknik, Langston, & Martinez-Conde, 2013; Schneider et al., 2021; Hung, Barbot, & Carrasco, 2023; Palmieri et al., 2023; Barquero, Chen, Munoz, & Wang, 2023), and appropriate to identify the slow-varying trends of interest.

We performed cluster-based permutation tests on the microsaccade rate time courses using MATLAB (Maris & Oostenveld, 2007), and implemented Bonferroni correction. When data were split based on preceding performance (Correct_N-1_ − Incorrect_N-1_), and no effect was observed in the Incorrect_N-1_ condition, we downsampled Correct_N-1_ trials to account for the possibility that the null effect in the Incorrect_N-1_ was due to the lower trial count. We randomly selected without replacement an equal number of Correct_N-1_ trials, computed the microsaccade rates, and performed cluster-based permutation tests (as described elsewhere in this article). We repeated this procedure 500 times per condition and calculated how frequently a significant cluster was detected within that condition.

#### Microsaccade timing analysis

Precise timings of the microsaccades were analyzed to investigate the sequential effects of foreperiod on temporal attention and expectation. We, therefore, focused on the microsaccade that happen around the stimulus presentation timing. To study pre-stimulus window microsaccade inhibition (Figure 1A), we analyzed the timing of the last microsaccade that occurred before the earliest possible target. We defined inhibition latency as the onset of the last microsaccade occurring in this pre-stimulus window. For post-stimulus microsaccade rebound, we analyzed the timing of the first microsaccade that occurred after the T1 onset regardless of the trial's target timing (Bonneh, Adini, & Polat, 2015; Denison et al., 2019). Trials without any microsaccade (13% across all conditions) were excluded from the timing analysis.

For each observer, we separately detected the inhibitory and rebound microsaccade. We then converted all of the microsaccade onset timings to *z*-scores, and estimated the kernel density for each pre-cue, expectation, and preceding foreperiod condition. Density was evaluated using 300 equally spaced points between −5 and 5, and the median of each kernel was computed to perform further statistical analyses. We used MATLAB for kernel estimation, and R for statistical tests.

## Results

### Behavior

The effectiveness of the Best parameter estimation by sequential testing algorithm was confirmed, with baseline performance for the Neutral condition yielding a μ = 72.746, SE = 1.577.

To investigate sequential effects of stimulus onset on the interaction between temporal expectation and attention, we conducted four-way ANOVAs (2 target × 2 cue validity × 3 current trial's onset (CTO) × 3 preceding foreperiod) analyzing discriminability (d′), criterion and RT. The experimental factors were target (T1 and T2), attentional cue validity (Valid and Neutral), CTO (Early_N_, Expected_N_, and Late_N_), and preceding foreperiod (Early_N-1_, Expected_N-1_, and Late_N-1_).

**Figure 2.**
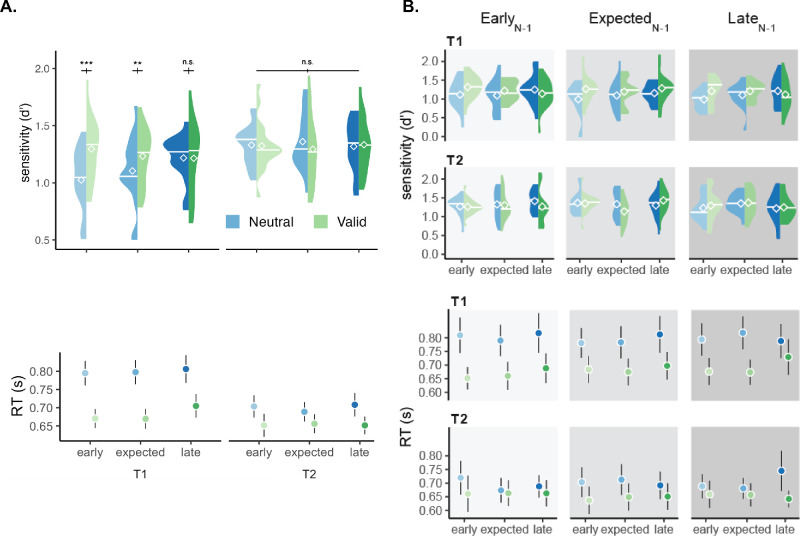
(**A**) The effects of target (T1, T2), cue validity (Valid, Neutral), and current trial's onset timing (EarlyN, ExpectedN, LateN) on sensitivity (d′) and reaction times (RTs). (**B**) Data split by preceding foreperiod. Preceding foreperiod is shown at different columns, highlighted by different shades of grey. Data from Duyar et al., 2024.

For discriminability (Figure 2) there were significant two-way interactions between target and cue validity, F(1,15) = 12.775, *p* = 0.003, *η_G_*^2^ = 0.013, as well as between cue validity and CTO, F(2,30) = 3.658, *p* = 0.038, *η_G_*^2^ = 0.005. Pairwise *t* tests following this interaction revealed a significant effect of cue validity, with higher discriminability in the Valid than the Neutral conditions in the Early (*p*_holm_ = 0.007, *d* = 0.313), but not at Expected (*p*_holm_ = 0.24), or Late time points (*p*_holm_ = 0.52), collapsed across target. These effects are all consistent with those in our previous study (Duyar et al., 2024). There were no main effects of the preceding foreperiod or interaction of this factor with other variables (all *p* > 0.1). All of these no significant effects were confirmed by a Bayes factor of less than 0.069 (<1 indicates an effect absence), except for the interaction of preceding foreperiod and target (2.641, <10 indicates a moderate effect).

For criterion, there were no significant main effects or interactions of any of the experimental factors (all *p*s > 0.1).

For RT (Figure 2), we found significant main effects of target, F(1,15) = 6.746, *p* = 0.020, *η_G_*^2^ = 0.023, and cue validity, F(1,15) = 17.513, *p* < 0.001, *η_G_*^2^ = 0.037. There was a two-way significant interaction between target and cue validity, F(1,15) = 17.263, *p* < 0.001, *η_G_*^2^ = 0.007. Pairwise comparisons for the interaction between target and cue validity revealed significant effects of cue validity for both targets, the faster RT in the Valid than the Neutral conditions was more pronounced for T1 (*p*_holm_< 0.001, *d* = 0.528) than T2 (*p*_holm_< 0.001, *d* = 0.238). There were no main effects of the preceding foreperiod or interaction of this factor with other variables (all *p* > 0.1).

To rule out the possibility that the non-significant preceding foreperiod could be due to insufficient statistical power in the four-way ANOVA, we conducted additional three-way ANOVAs. First, we removed the factor “target” (2 cue validity × 3 CTO × 3 preceding foreperiod). For discriminability, there was an interaction between the cue validity and CTO, F(2,30) = 4.625, *p* = 0.017, *η_G_*^2^ = 0.012, but no main effect of the preceding foreperiod or interaction of this factor (all *p* > 0.1). For RT, there were main effects of cue validity, F(1,15) = 17.167, *p* = 0.0008, *η_G_*^2^ = 0.0044, and CTO, F(2,30) = 6.010, *p* = 0.006, *η_G_*^2^ = 0.0015, but no main effect of preceding foreperiod or interaction of this factor (all *p* > 0.1). Second, we computed the difference between the Neutral and the Valid conditions (2 target × 3 CTO × 3 preceding foreperiod). For discriminability, there were main effects of target, F(1,15) = 11.864, *p* = 0.003, *η_G_*^2^ = 0.032, and CTO, F(2,30) = 3.413, *p* = 0.046, *η_G_*^2^ = 0.012, but no main effect of preceding foreperiod or interaction of this factor (all *p* > 0.1). For RT, there was a main effect of target, F(1,15) = 16.827, *p* = 0.0009, *η_G_*^2^ = 0.081, but no main effect of the preceding foreperiod or interaction with this factor (all *p* > 0.1).

Splitting the data based on preceding performance confirmed that there was no effect of the preceding foreperiod on discriminability (all main effects and interactions with preceding foreperiod, *p* > 0.1). Consistent with the overall analyses, in Correct_N-1_ trials there was an interaction between cue validity and target, F(1,15) = 14.443, *p* = 0.002, *η_G_*^2^ = 0.015, and a marginal two-way interaction between cue validity and CTO, F(2,30) = 3.106, *p* = 0.059, *η_G_*^2^ = 0.007. In Incorrect_N-1_ trials, there was an interaction among target, pre-cue and CTO, F(2,30) = 11.167, *p* = 0.0002, *η_G_*^2^ = 0.020.

In sum, for performance, there were main effects of cue validity and an interaction between cue validity and CTO, resulting in higher attentional benefits for earlier than expected targets. However, there was no main effect of the preceding foreperiod or interaction with other factors, regardless of whether the previous trial was correct or incorrect, indicating that attentional modulations on visual performance are not updated based on the timing of the preceding trial.

### Microsaccade rate

**Figure 3.**
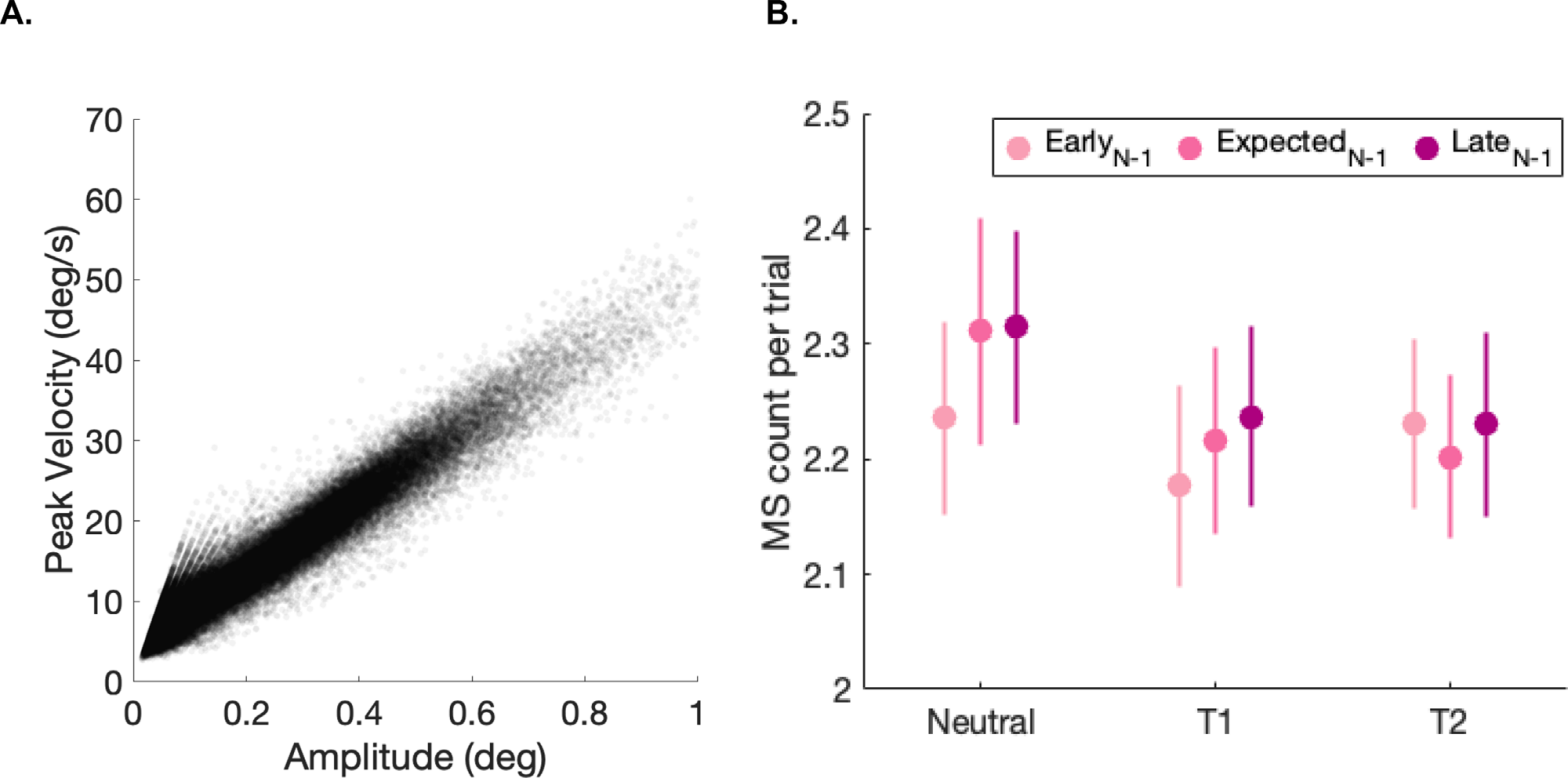
(**A**) Microsaccade (MS) main sequence, peak velocity vs amplitude. Each point represents a MS. (**B**) Total number of MSs, normalized by the trial count, split by attention and preceding foreperiod condition.

We confirmed that peak velocity and amplitude were correlated (R^2^ = 0.913) (Figure 3A). For completeness, we also plot the total number of microsaccade per trial separated by attention and expectation condition (Figure 3B). We analyzed the microsaccade rates in the pre-stimulus window, defined as the period between the pre-cue and 1,200 ms, which is the earliest possible stimulus onset (Figure 1A).

**Figure 4.**
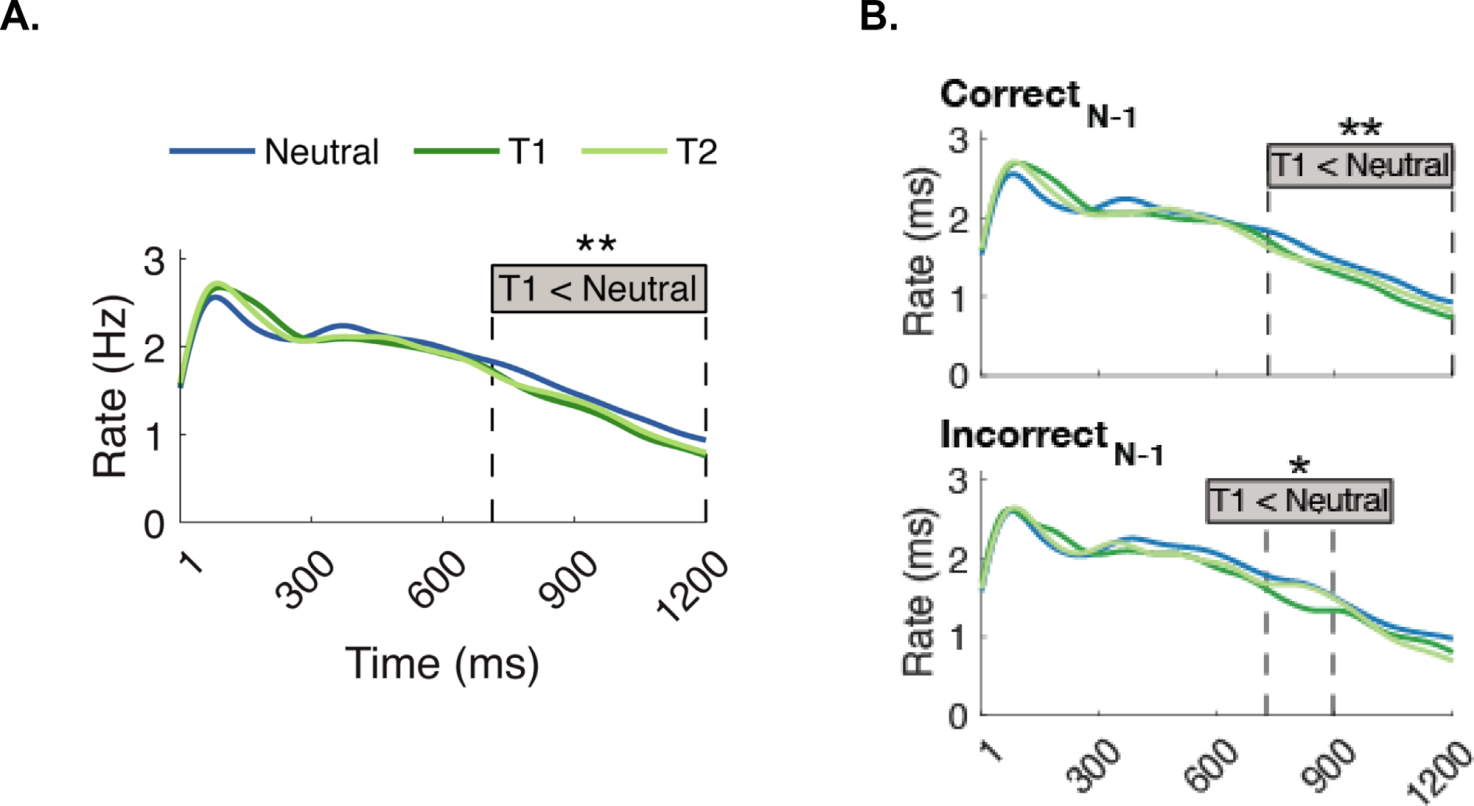
Effects of the attentional pre-cue on microsaccade rates in the pre-stimulus window. (**A**) Effects of the attentional pre-cue regardless of preceding foreperiod. Microsaccade rates in T1-cued trials were significantly lower than in neutral trials between 713 and 1,200 ms. (**B**) Attentional effects based on preceding performance (Correct_N-1_: 731–1,200 ms; Incorrect_N-1_: 726–895 ms).

To investigate attentional effects in this time window, we first compared microsaccade rates within each attentional pre-cue condition, regardless of the preceding foreperiod (Figure 4A). Microsaccade rates in the T1-cued condition were significantly lower than in the Neutral condition between 713 and 1,200 ms, 487 ms duration, *p*_Bonferroni_ = 0.003, Neutral(μ, SE) = (1.369, 0.088), T1(μ, SE) =(1.202, 0.086).

We split data based on preceding performance (Figure 4B). Microsaccade rates in the T1-cued condition were lower than in the Neutral condition in both Correct_N-1_ (between 731 and 1,200 ms, 469 ms duration, *p*_Bonferroni_ = 0.017) and Incorrect_N-1_ (between 726 and 895 ms, 169 ms duration, *p*_Bonferroni_ = 0.042).

**Figure 5.**
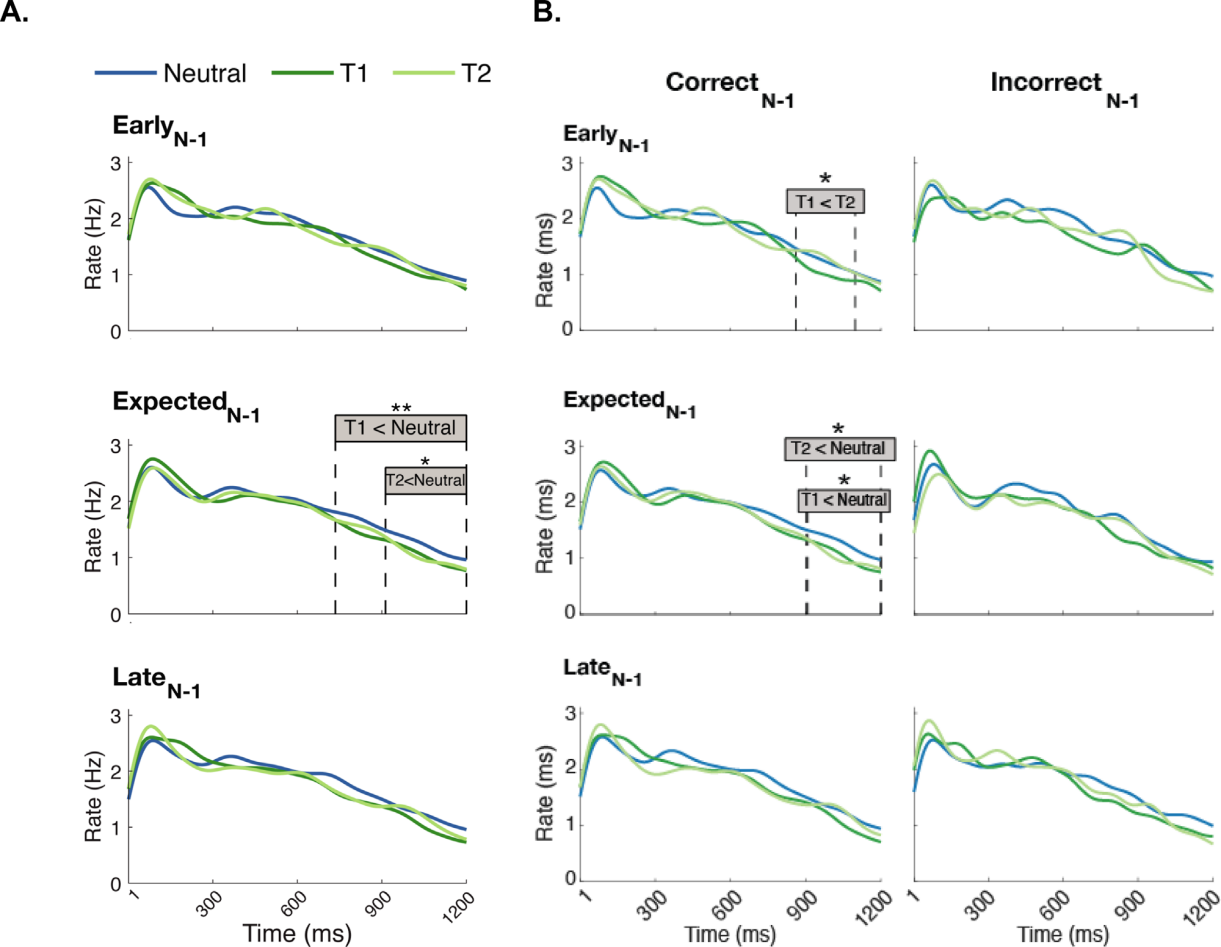
Effects of the attentional pre-cue on microsaccade rates within each preceding foreperiod condition. (**A**) In the Expected_N-1_ trials, neutral trials showed significant differences in microsaccade rates compared with T1-cued trials between 735 and 1,200 ms and T2-cued trials between 913 and 1,200 ms. (**B**) In Correct_N-1_ trials (left), attentional effects emerged in Early_N-1_ (lower microsaccade rates in T1-cued than T2-cued trials between 861-1098 ms), as well as in Expected_N-1_, where we found lower microsaccade rates in T1-cued (908–1,200 ms) and T2-cued (903–1,200 ms) trials than Neutral. No attentional effect emerged in Incorrect_N-1_ trials (right).

We investigated potential effects of preceding foreperiods on the attentional modulation on microsaccade rates, by comparing the effect of attentional pre-cues (T1, T2, and Neutral) on microsaccade rates as a function of the preceding foreperiod (Early_N-1_, Expected_N-1_, and Late_N-1_) (Figure 5A). There were two significant clusters in the Expected_N-1_ condition (middle), but not in the two other preceding foreperiods. Microsaccade rates in the Neutral trials were higher than the rates in T1-cued trials between 735 and 1,200 ms, 465 ms duration, *p*_Bonferroni_ = 0.003, Neutral(μ, SE) = (1.388, 0.095), T1(μ, SE) = (1.187, 0.095), and than in T2-cued trials between 913 and 1200 ms, 287 ms duration, *p*_Bonferroni_ = 0.038, Neutral(μ, SE) = (1.213, 0.094), T2(μ, SE) = (1.006, 0.095).

We also assessed these effects as a function of preceding performance (Figure 5B). In Correct_N-1_ trials, attentional modulations were found in Early_N-1_ (Lower microsaccade in T1- than T2-cued trials between 861 and 1,098 ms, 237 duration, p_Bonferroni_ = 0.023), Expected_N-1_ (Lower microsaccade in T1-cued than Neutral: between 908 and 1,200 ms, 292 ms duration, *p*_Bonferroni_ = 0.042, and in T2-cued than Neutral trials between 903 and 1,200 ms, 297 ms duration, *p*_Bonferroni_ = 0.011). In the Incorrect_N-1_ trials, no significant clusters emerged in any of the comparisons. When we downsampled Correct_N-1_, in Early_N-1_, T1–T2 difference emerged in 9.6% of the repetitions; in Expected_N-1_, T1–Neutral difference emerged in 7.4% of the repetitions, and T2–Neutral difference emerged in 5.8% of the repetitions.

**Figure 6.**
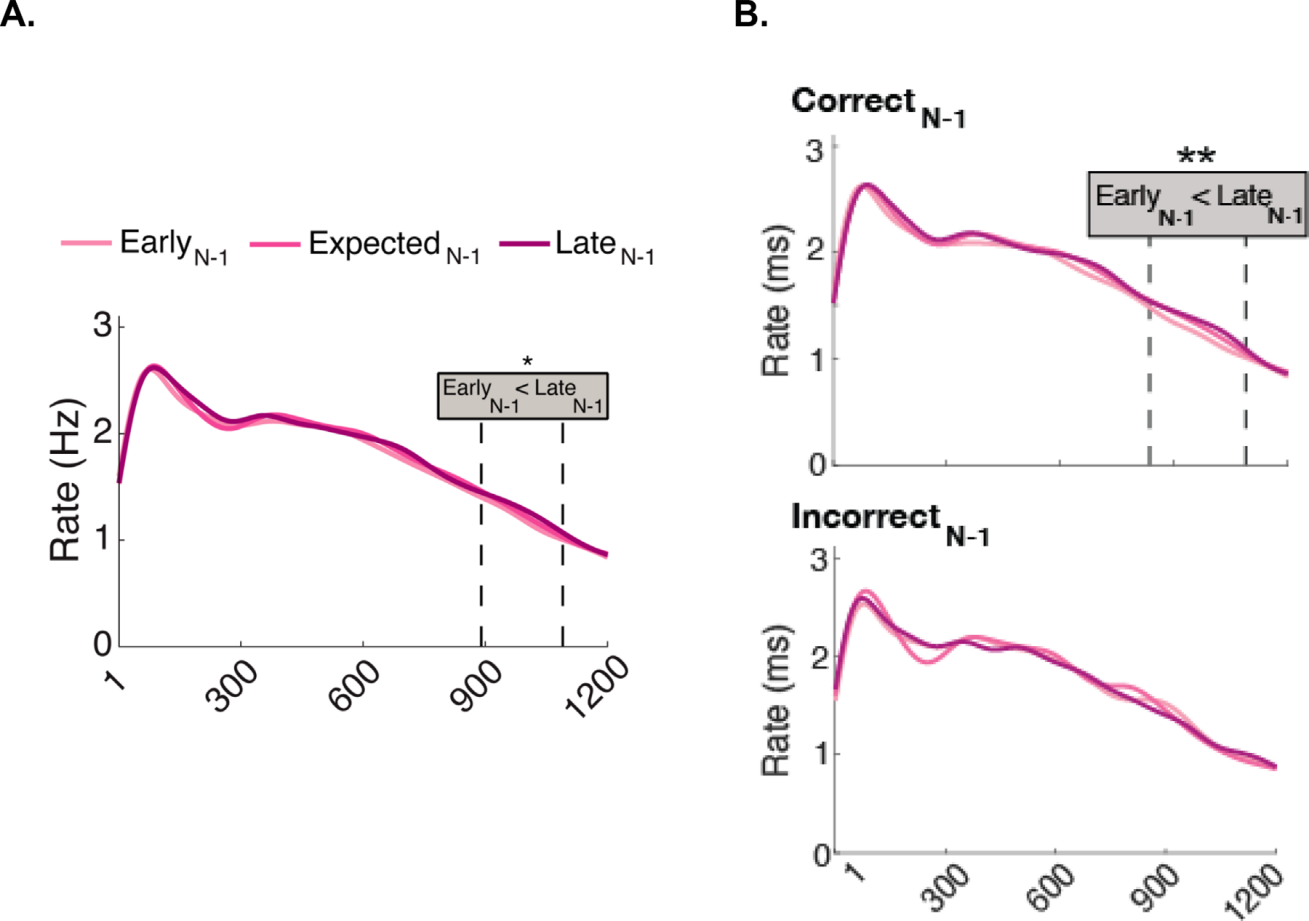
The effect of the preceding foreperiods (Early_N-1_, Expected_N-1_, and Late_N-1_) on microsaccade rates regardless of attentional pre-cue. (**A**) Early_N-1_ and Late_N-1_ conditions differed between 890 and 1,090 ms. (**B**) In Correct_N-1_ trials, the Early_N-1_ and Late_N-1_ conditions differed between 836 and 1,090 ms. No effect of preceding foreperiod emerged in Incorrect_N-1_ trials (bottom).

We then investigated the potential effects of preceding foreperiod in the pre-stimulus window, regardless of the attentional pre-cue (Figure 6A). Microsaccade rates were significantly lower in trials Early_N-1_ than Late_N-1_ between 890 and 1,090 ms (200 ms duration, *p*_Bonferroni_ = 0.046).

We then investigated whether preceding performance modulated the effects of preceding foreperiod, regardless of the attentional pre-cue (Figure 6B). In Correct_N-1_ trials, microsaccade rates were lower in Early_N-1_ than Late_N-1_ trials between 836 and 1,090 ms (254 ms duration, *p*_Bonferroni_ = 0.006). No significant clusters emerged in the Incorrect_N-1_ trials. When we downsampled Correct_N-1_, Early_N-1_–Late_N-1_ difference emerged in 63.8% of the repetitions.

**Figure 7.**
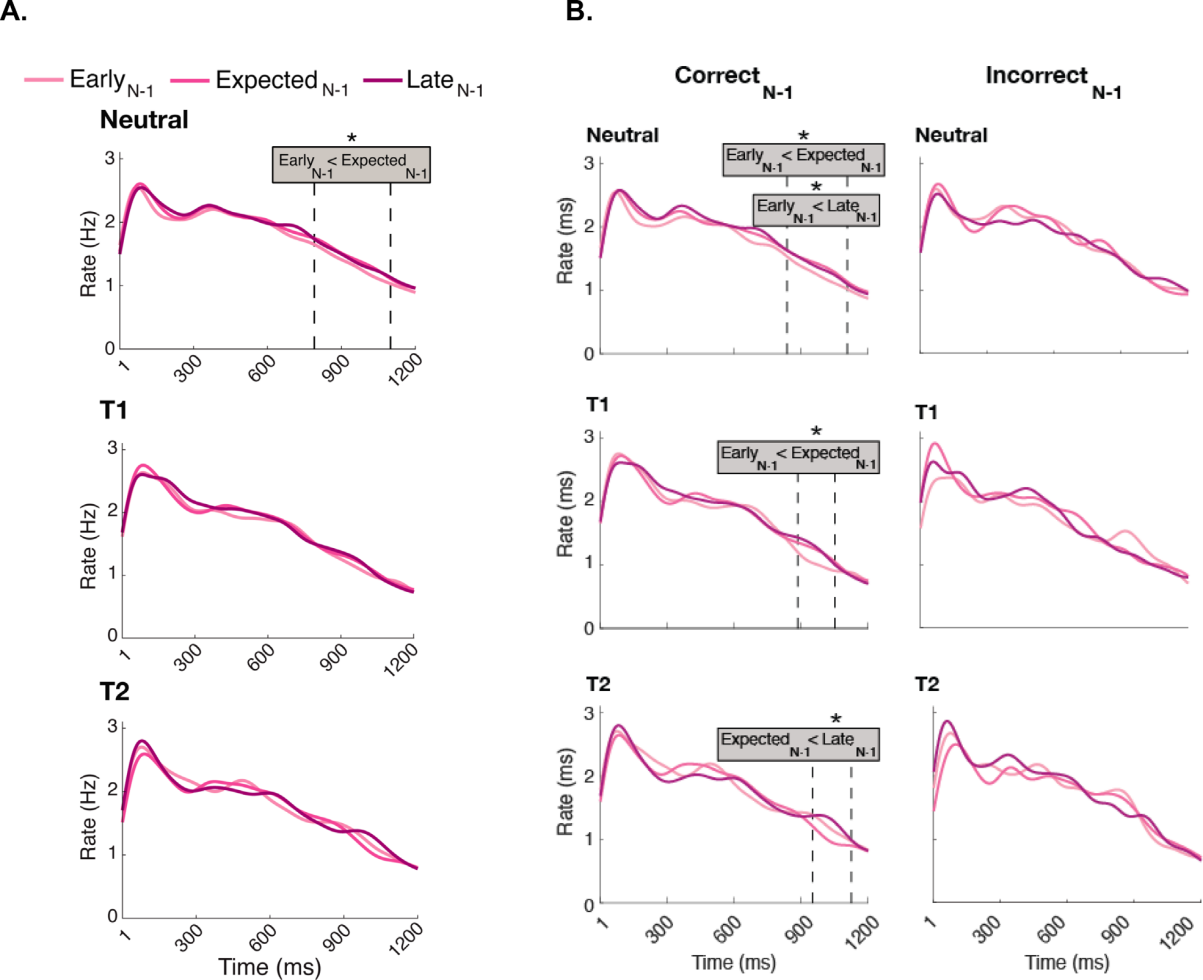
The effect of the preceding foreperiod on microsaccade rates within each attentional pre-cue (Neutral, T1, and T2) condition. (**A**) In the Neutral condition, a significant difference was observed in the 790- to 1,099-ms interval, highlighting when Early_N-1_ and Expected_N-1_ conditions diverge. No significant effects were found in either T1 or T2 pre-cue conditions. (**B**) In Correct_N-1_ trials (left), significant differences emerged among preceding foreperiod in all attention conditions. In Neutral trials, microsaccade (MS) rates were lower in Early_N-1_ than Expected_N-1_ (between 838 and 1,107 ms) and than Expected_N-1_ (between 837 and 1,107 ms). In T1-cued trials, MS rates were lower in Early_N-1_ than Expected_N-1_ (between 886 and 1,051 ms). In T2-cued trials, MS rates were lower in Expected_N-1_ than Late_N-1_ (between 952 and 1,124 ms). No preceding foreperiod effect emerged in Incorrect_N-1_ trials (right).

To investigate whether the effects of preceding foreperiod vary with attention, we compared across microsaccade rates within each attentional pre-cue condition as a function of the preceding foreperiod (Early_N-1_, Expected_N-1_, and Late_N-1_). A significant cluster was detected only in the Neutral condition (top), where microsaccade rates were lower in Early_N-1_ than Expected_N-1_ between 790 and 1,099 ms after the pre-cue (309 ms duration, *p*_Bonferroni_ = 0.012) (Figure 7A); no significant cluster emerged in the T1 and T2 attentional pre-cue conditions.

We assessed whether these effects varied with preceding performance (Figure 7B). In Neutral trials, microsaccade rates were lower in Early_N-1_ than Expected_N-1_ between 838 and 1,107 ms (269 ms duration, *p*_Bonferroni_ = 0.015), and Late_N-1_ between 837 and 1,107 ms (837 ms duration, *p*_Bonferroni_ = 0.030). In T1-cued trials, microsaccade rates were lower in Early_N-1_ than Expected_N-1_ between 886 and 1,051 ms (165 ms duration, *p*_Bonferroni_ = 0.049). In T2-cued trials, microsaccade rates were lower in Expected_N-1_ than in Late_N-1_ between 952 and 1,124 ms (172 ms duration, *p*_Bonferroni_ = 0.023). No significant clusters emerged in the Incorrect_N-1_ trials. When we downsampled Correct_N-1_, in Neutral, Early_N-1_-Expected_N-1_ and Early_N-1_-Late_N-1_ differences emerged in 0.2% and 1.4% of the repetitions, respectively. In T1-cued trials, Early_N-1_–Expected_N-1_ difference emerged in 0.4% of the repetitions. In T2-cued trials, Expected_N-1_–Late_N-1_ differences emerged in 0.6% of the repetitions.

In sum, microsaccade rates during the pre-stimulus period were lower in the attention than in the neutral trials, and in Early_N-1_ than in the Expected_N-1_ trials. Cluster-based permutation tests revealed attentional effects in Expected_N-1_ and preceding foreperiod effects in the Neutral trials. Furthermore, attention modulated microsaccade rates regardless of the preceding foreperiod, and the preceding foreperiod modulated microsaccade rates regardless of attention only when performance was correct in the preceding trial.

### Microsaccade timing

**Figure 8.**
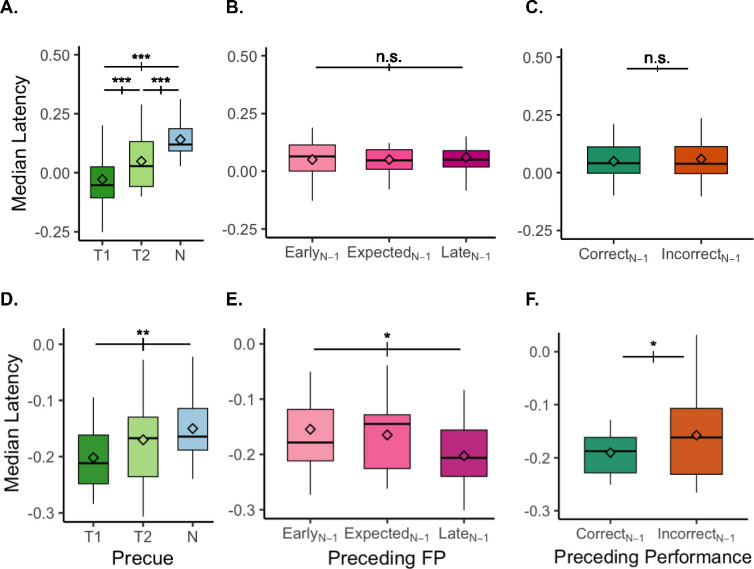
Microsaccades timing around stimulus onset. Inhibition of microsaccades was analyzed within the pre-stimulus window (from pre-cue onset to 1,200 ms), and quantified as the latency of the last pre-T1 microsaccade (**A**–**C**). The rebound was quantified as the latency of the first post-T1 Microsaccade (**D**–**F**). (**A**) Last pre-T1 microsaccade (MS) was executed earlier with attention. (**B**) Last pre-T1 MS latency was similar regardless of the preceding foreperiod. (**C**) Last pre-T1 MS latency was similar regardless of preceding performance. (**D**) First post-T1 MS was executed earlier with attention. (**E**) First post-T1 MS was executed earlier for Late preceding foreperiod. (**F**) First post-T1 MS was executed earlier when the preceding performance was correct. n.s., not significant.

Three-way ANOVAs (3 preceding foreperiod × 2 preceding performance × 3 pre-cue) were conducted to analyze their potential timing effects on pre-stimulus inhibition and post-stimulus rebound. Pre-stimulus inhibition was modulated only by pre-cue (Figure 8A), F(2,30) = 10.868, *p* = 0.0002, *η_G_*^2^ = 0.137. It was earlier in T1-cued than T2-cued (*p*_holm_ = 0.0008, *d* = 0.402), which was in turn earlier than Neutral trial (*p*_holm_ = 0.0007, *d* = 0.538). There was no effect of preceding foreperiod (Figure 8B) or preceding performance (Figure 8C) on inhibition latency. Post-stimulus rebound latency was modulated by pre-cue (Figure 8D) F(2,30) = 4.663, *p* = 0.017, *η_G_*^2^ = 0.025, it was earlier in T1-cued than Neutral (*p*_holm_ = 0.005, *d* = 0.396), preceding foreperiod (Figure 8E) F(2,30) = 5.134, *p* = 0.012, *η_G_*^2^ = 0.024, it was earlier in Late_N-1_ than Early_N-1_ (*p*_holm_ = 0.031, *d* = 0.348), and preceding performance (Figure 8F) F(1,15) = 6.555, *p* = 0.022, *η_G_*^2^ = 0.015, it was earlier in Correct_N-1_ than Incorrect_N-1_ (*p*_holm_ = 0.031, *d* = 0.348). There was no interaction among these factors (all *p* > 0.1).

## Discussion

Temporal expectation, the process that enables us to predict the onset of an upcoming event, and attention, the process that enables us to select task-relevant specific time points, facilitate the allocation and distribution of limited resources. In this study, we investigated the flexibility of attention under temporal uncertainty on behavioral performance and oculomotor dynamics. We reanalyzed data from our previous study (Duyar et al., 2024), which showed that temporal uncertainty results in high attentional benefits on performance at earlier-than-expected moments, which gradually decrease with target delay. We assessed the potential role of preceding foreperiods on attentional deployment over time.

We found that the preceding foreperiod does not modulate the effects of attention on either behavioral performance or microsaccade timing, but it does modulate microsaccade rates. These findings indicate that microsaccade timing is related to stable attentional processes that govern visual performance, whereas microsaccade rates are influenced by dynamic factors, such as the varying expectations from trial to trial. Therefore, although the perceptual system represents the preceding foreperiod in oculomotor dynamics, it does not affect the specific time when attention benefits performance. These findings, as well as a recent study showing that serial dependence is manifested in perceptual performance but not in pupil responses (Guan & Goettker, 2024), are consistent with the different goals of the perceptual system—to integrate sensory information to create a stable percept across time—and the oculomotor system—to maintain foveation. Moreover, they provide further evidence of perception-eye movement dissociations (Carrasco & Spering, 2024; Spering & Carrasco, 2015).

The effects of attention on performance were stable (Figure 2). This finding is noteworthy given that many perceptual judgements are biased toward previous stimuli in visual (Cicchini, Anobile, & Burr, 2014; Cicchini, Mikellidou, & Burr, 2023; Fischer & Whitney, 2014; Pascucci et al., 2023; Taubert, Van der Burg, & Alais, 2016), auditory (Motala et al., 2020), and olfactory (van der Burg et al., 2022) modalities. The preceding foreperiod affects the speed of perceptual judgements (Capizzi et al., 2015; Possamai, Granjon, Requin, & Reynard, 1973; Steinborn & Langner, 2012; Tal-Perry & Yuval-Greenberg, 2022). RTs decrease when the current foreperiod is shorter than the preceding foreperiod, indicating that expectation can be flexibly updated on a trial-by-trial basis. Given the established effects of trial sequence in perceptual judgments and response time, we hypothesized that the preceding foreperiod could affect allocation of attention under temporal uncertainty. However, we found that the performance benefits of attention under uncertainty remained consistent regardless of the preceding foreperiod (Figure 2). This finding indicates that control of attention is not updated based on the timing of individual trials, and suggests that observers aim to optimize their overall visual performance.

The pre-cue evokes a reflexive inhibition in microsaccade rate (Engbert & Kliegl, 2003; Rolfs, Engbert, & Kliegl, 2005) and a persistent decrease during the anticipatory period (Abeles et al., 2020; Amit et al., 2019; Badde et al., 2020; Betta & Turatto, 2006; Dankner et al., 2017). Temporal attention deepens this anticipatory reduction beyond mere expectation (Denison, Yuval-Greenberg, & Carrasco, 2019; Palmieri et al., 2023). Both of these studies investigated temporal attention with no temporal uncertainty, keeping the stimulus timing constant to control for expectation. Consistent with these studies, here we found that, during the anticipatory period, reduction was amplified for an attended specific moment compared with the Neutral condition (Figure 4), and that this deepening in anticipatory reduction persists even under temporal uncertainty (Figure 4A), and regardless of preceding performance (Figure 4B). Reduction of microsaccade has been linked to preservation of perceptual processing resources (Badde et al., 2020). In line with this account, temporal attention may facilitate the preservation of these resources to be allocated at a specific moment when a task-relevant stimulus appears.

We also investigated whether the preceding foreperiod modulates attention effects on microsaccade rate in the pre-stimulus window. Attentional strengthening depends on the preceding foreperiod: Attention facilitates pre-stimulus rate inhibition only in trials that follow a trial with expected timing (Figure 5A). Such an effect, which provides further evidence to the top-down nature of endogenous temporal attention, was present when preceding performance was correct, but not when incorrect (Figure 5B).

With regard to expectation, pre-stimulus inhibition was stronger for Early than Late preceding foreperiods across attention conditions (Figure 6A). This pattern was observed in neutral trials (Figure 7A, top), consistent with a recent finding on expectation (Tal-Perry & Yuval-Greenberg, 2023). Interestingly, when observers were instructed to selectively attend to T1 or T2, the preceding foreperiod effect did not emerge (Figure 7A, middle and bottom). These findings indicate that attention overrides the preceding foreperiod effect that occurs with expectation.

The effect of preceding foreperiod was observed in all attentional conditions when the trial was preceded by a correct response (Figure 7B). However, it was strongest in Neutral trials (top), where we identified two clusters with a longer duration than in T1-cued and T2-cued trials (middle and bottom). The presence of additional clusters in Correct_N-1_ trials (Figure 7B), compared with the overall analysis (Figure 7A), suggests that the preceding foreperiod may be more reliable when the preceding performance was correct. However, we note that the absence of significant clusters in Incorrect_N-1_ trials (Figures 5^6^–7B) is likely due to lower number of incorrect responses in the experiment, resulting in fewer trials for analysis. When we downsampled the Correct_N-1_ trials, they were still significant in some cases (Early_N-1_ < Late_N-1_) (Figure 6B), but not others, indicating absence of evidence rather than evidence of absence for the Incorrect_N-1_ trials (Figures 5B, 7B). Thus, we can only conclude that preceding performance mattered for the overall preceding foreperiod effect (Figure 6B).

Attention modulated the precise timing of microsaccade around the target presentation: It shifted both the last microsaccade before the stimulus onset (Figure 8A) and the first microsaccade after the stimulus offset earlier (Figure 8D). These findings agree with those in a similar study, but in which T1 and T2 always occurred with predictable timings (Denison et al., 2019). Notably, this earlier suppression and rebound were present under temporal uncertainty and were independent of the preceding foreperiod and preceding performance. The earlier suppression in the pre-stimulus window, helping gaze stabilization, seems to be resilient to varying temporal dynamics (Figure 8B) and immediate response history (Figure 8C). In contrast, the first post-T1 microsaccade latency was influenced both by the preceding foreperiod and preceding performance. It was earlier when the preceding foreperiod was Late (Figure 8E), and when the preceding performance was Correct (Figure 8F). These effects were independent from temporal attention, indicating that observers did not adjust their strategy based on immediate trial history. The earlier rebound after Late trials might be a residual effect from prolonged microsaccade inhibition in the preceding trial, whereas the earlier rebound after correct trials suggests that a successful response facilitates a quicker return to baseline motor behavior in subsequent trials.

In the current study, temporal attention benefitted visual performance at earlier than expected moments, regardless of the preceding trial's timing. Thus, the temporal selection history—the specific moment observers attended in the preceding trial—did not guide attention in the subsequent trial at the performance level. Although “selection history” has been considered as a distinct type of involuntary attentional control (Awh, Belopolsky & Theeuwes, 2012), there are mixed results regarding selection history effects on attentional deployment (e.g., Choe & Kim, 2022; Kristjánsson & Ásgeirsson, 2019; Pascucci et al., 2023; Ramgir & Lamy, 2021). For instance, selection history modulates the deployment of pre-saccadic attention (White, Rolfs, & Carrasco, 2013), whereas in visual search, it does not influence stimulus-driven spatial attention (Yashar, White, Fang, & Carrasco, 2017).

## Conclusions

Visual performance and oculomotor responses can be dissociated under temporal uncertainty. Attention allocation is independent from the preceding foreperiod for both behavioral performance and precise timing of microsaccade, but not for microsaccade rates.

Our finding of consistent behavioral attentional deployment regardless of temporal selection history suggests that although selection history can be used as one of the sources of expectation in subsequent trials, it does not necessarily determine, strengthen, or guide attentional deployment. Our finding that preceding temporal experiences alter microsaccade rates, suggest that microsaccade rates are not a reliable index of temporal attention allocation or of its benefits on visual performance.
